# Airborne metofluthrin, a pyrethroid repellent, does not impact foraging honey bees

**DOI:** 10.1093/jisesa/ieae103

**Published:** 2024-10-23

**Authors:** Margaret J Couvillon, John Hainze, Connor Bizon, Lindsay E Johnson, Ian F McKellips, Benjamin E McMillan, Bradley D Ohlinger, Robert B J Ostrom, Roger Schürch

**Affiliations:** Department of Entomology, Virginia Tech, Blacksburg, VA, USA; Department of Civil and Environmental Engineering, Seattle University, Seattle, WA, USA; Thermacell Repellents Inc., Bedford, MA, USA; Department of Entomology, Virginia Tech, Blacksburg, VA, USA; Department of Entomology, Virginia Tech, Blacksburg, VA, USA; Thermacell Repellents Inc., Bedford, MA, USA; Department of Entomology, Virginia Tech, Blacksburg, VA, USA; Odum School of Ecology, University of Georgia, Athens, GA, USA; Department of Entomology, Virginia Tech, Blacksburg, VA, USA; Department of Entomology, Virginia Tech, Blacksburg, VA, USA

**Keywords:** metofluthrin, foraging, waggle dance, bee communication

## Abstract

Outdoor spatial mosquito repellents, such as mosquito coils or heating devices, release pyrethroid insecticides into the air to provide protection from mosquitoes within a defined area. This broadcast discharge of pyrethroids into the environment raises concern about the effect on non-target organisms. A previous study found that prallethrin discharged from a heating device did not affect honey bee (*Apis mellifera* L.) [Hymenoptera: Apidae] foraging or recruitment. In this second study, there was no significant difference in foraging frequency (our primary outcome), waggle dance propensity, or persistency in honey bees collecting sucrose solution between those exposed to metofluthrin from a different heating device and bees exposed to a non-metofluthrin control. One measure, waggle dance frequency, was higher in the metofluthrin treatment than the control but this outcome was likely a spurious result due to the small sample size. The small particle size of the emissions, averaging 4.43 µm, from the heated spatial repellent products, which remain airborne with little settling, may play an important role in the lack of effect found on honey bee foraging.

## Introduction

Pyrethroid insecticides are used broadly in agricultural and domestic settings to control organisms such as ectoparasites (Taplin and Meinking 1990), including mosquitoes (Churcher et al. 2016) and arthropods affecting agricultural crops (Joseph et al. 2017). The environmental and non-target effects of these active ingredients vary as a result of differences in chemical composition (Ujihara 2019). For example, the chemical structure of permethrin or deltamethrin resists breakdown in the environment and provides longer lasting, residual toxicity, which may be important for agricultural or domestic pest control. Other pyrethroids break down more quickly in the environment, including the naturally occurring pyrethrins and pyrethroids from the allethrin series (Matsuo 2019). All these ingredients, residually active or not, are toxic in sufficient quantity and with direct contact, to all arthropods. Yet, pyrethroid insecticides also have sub-lethal effects, with repellency being mentioned most frequently with respect to mosquitoes (Achee et al. 2009).

Spatial repellents are a category of mosquito repellents that may be used indoors and outdoors and have recently generated interest for their application in prevention of mosquito-borne diseases such as dengue fever and malaria (Achee et al. 2023). Pyrethroid repellency of mosquitoes has been demonstrated outdoors, but researchers also speculate that other effects may result from airborne contact with pyrethroids (Bibbs and Kaufman 2017). While indoor use would appear to have little environmental effect, outdoor use of spatial repellents raises questions about effects on non-target species. Recently, it was found that prallethrin dispensed as a spatial repellent did not influence honey bee (*Apis mellifera*) foraging and recruitment behavior (Couvillon et al. 2023). This study examines the effect of a second pyrethroid dispensed as a spatial repellent, metofluthrin, on honey bee behavior and speculates on the influence of new information on the physical chemistry of spatial repellent emissions.

Honey bees are of global importance as pollinators and are often used to represent the effects of chemicals on bees more broadly (regulated in 40 CFR 158; see Cresswell 2011 for a meta-analysis). Training bees to frequent an artificial feeder (Couvillon et al. 2015) allows researchers to assess foraging and recruitment for treatment effects. Previously, this approach was used to evaluate the effects of caffeine, which increases foraging and recruitment (Couvillon et al. 2015), and the neonicotinoid imidacloprid, which decreases activity at the feeder (Ohlinger et al. 2022a). However, there is limited evidence whether the presence of a nearby pest control device (like E90 Rechargeable Mosquito Repeller) that emits volatiles might impact honey bee behaviors (Couvillon et al. 2023). More work is needed to examine how the bees might behave in the presence of different pesticides.

Here, we investigated honey bee foraging and recruitment in the presence of the pyrethroid metofluthrin. Specifically, we examine the effect of the heated spatial repellent on honey bee foraging frequency, waggle dance frequency, waggle dance propensity, and persistency in a field experiment with freely flying bees.

## Materials and Methods

### Experimental Design

For this study, we used 3 honey bee colonies (*Apis mellifera ligustica*) housed in glass-walled observation hives at a research station in Blacksburg, Virginia. We established the observation hives inside our bee lab, and each hive contained 3 American Standard Deep frames, and connected the hives to the outside via a 3 × 30 cm plastic tube. We worked with one colony at a time and removed each colony after the completion of the trial. We managed the colonies to prevent overcrowding and to standardize the number of empty cells to provide space for additional sucrose solution storage (Seeley 1989, 1995, Couvillon et al. 2015). No supplemental food was provided during the field experiments.

**Fig. 1.**
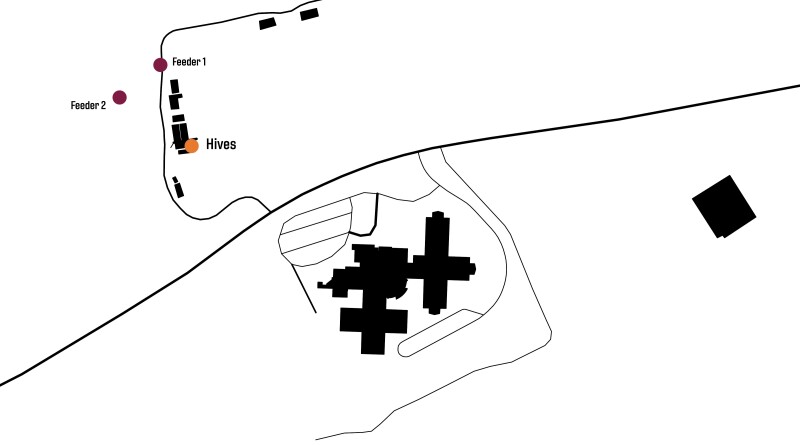
Field trial set-up at our Research Station. We worked with 3 colonies in series, one per trial. We trained 2 cohorts of bees to forage from one of two feeders that were identical and equidistant from the colony.

We established control and treatment feeder stations 100 m from the hive (Fig. 1). Each station contained a honey bee feeder and a repellent dispensing device from Thermacell Repellents, Incorporated. One device dispensed a treatment while the other was a control (see below), although they appeared identical—and therefore blinded—to field researchers. We placed honey bee feeders on a wooden platform atop a tripod at a height of 1 m. We placed the Thermacell device on a separate wooden platform at a height 1 m atop a tripod, 3.05 m from the feeder and perpendicular to the flight path of the bees. We arrived at the distance of 3.05 m based on Thermacell data (unpublished) provided to the US Environmental Protection Agency (EPA) to support product registration, demonstrating that mosquito repellency occurs at that distance.

We conducted 3 randomized, paired, parallel group trials (one per experimental day—designated as Day 0) on June 9, June 15, and June 29, 2023 at Prices Fork Research Station (Fig. 1). The timing coincided with reduced amounts of forage in the area for honey bees, which made it easier to train bees to feeders (Ohlinger et al. 2022b).We achieved an approximate 1:1 allocation ratio between treated (exposed to active compound at feeder) and control bees (not exposed at feeder). Thermacell provided blinded treatments, labeled as GAMMA and DELTA, for the investigators. Unblinding of investigators occurred on 23 October 2023, after the primary analysis had been performed. GAMMA was the control, and DELTA was the treatment containing the active ingredient metofluthrin.

On Day −1, we trained honey bees from the observation hives to forage from feeders at stepped, increasing distances (Ohlinger et al. 2022a, Couvillon et al. 2023). Normally, the training takes place on a single day but, because of weather conditions, it required as many as 3 training days prior to the experiment day for some trials in this study. On the days prior to the experiment, we placed a feeder containing a scented 2M sucrose solution on the wooden platform supported by a tripod approximately 1 m away from the hive entrance tube. The sucrose solution incorporated a peppermint scent in trial 1, a lavender scent in trial 2, and a linalool scent in trial 3, at a scent concentration of 10 µl/L sucrose solution. As bees accumulated on the feeder, we moved the feeder about 8–10 m on 10–12 successive occasions, across an adjacent parking lot. We marked bees at the feeder with individually numbered plastic discs (Queen Number Set, Betterbee, Greenwich, NY), allowing for individual identification and also confirmation that bees returned to the correct focal colony. This marking continued throughout the training phase (Days −1, −2, −3 and morning of Day 0). With each step, the trained bees imbibed from the feeder at least 3 times. It took a few hours, spread over several days depending on weather conditions, to move the feeders to a location directly in front of the 2 experimental feeders for the experimental day (“Feeder 1” and “Feeder 2”, Fig. 1).

We returned the tripod and feeder to the same ending position the morning of the experiment (Day 0) and reactivated the colony by squirting the same scented, 2M solution used on the prior days into the hive. The familiar scent induces the experienced foragers to return to the feeder (Reinhard et al. 2004). We placed an identical second feeder on the tripod platform and permitted the trained bees to self-sort between the 2 feeders. We then moved the 2 feeders away from each other, in successive steps, until they reached the final positions equidistant from the hive (Fig. 1). At that point, we commenced recording the identity and time of bee visits to each feeder on an Android tablet using the app ODK Collect. We uploaded the visitation report forms from ODK Collect into a Google sheet, which was stored on a Google Team Drive.

We discouraged recruitment from other hives located in our nearby apiary by removal of marked foragers that were not then confirmed in the observation hive. We left the feeders in place until at least 10 confirmed foragers were consistently visiting each feeder. This generally occurred mid-to-late afternoon on Day 0, immediately preceding the start of the experimental phase.

We used the code names “blue” and “yellow” for the 2 feeder locations. Blinded to the actual treatments, we randomized product refill vials, labeled GAMMA and DELTA, to the blue feeder with the yellow feeder getting the remaining treatment. The randomization for the blue feeder was GAMMA, GAMMA, and DELTA for the 3 trials (full randomization plan in Supplementary Data).

Sample sizes depend on bee recruitment and could not be controlled completely. Despite this constraint, we observed relatively even treatment allocations within trials and for the overall experiment (Treatment = 67 bees, Control = 74 bees). Correlation among bees within hives is very low for many behaviors and comparable to between-hive correlations (Schürch et al. 2016, 2019). Therefore, we consider each bee as an independent replicate. The random assignment of the treatment balances the treated/untreated devices feeder position.

### Experimental Trial Procedure

At the beginning of the experimental phase at 15:00 on Day 0, we replaced the feeders containing 2M scented syrup with 2 new feeders that contained 1M unscented solution. This concentration generates good (Ohlinger et al. 2022b), but not maximum, foraging and recruitment (Seeley 1995) in the study site during the time of year in which our work was conducted. We placed a Thermacell device, containing either a treatment or control refill, on a tripod 3.05 m from each feeder perpendicular to a direct line between hive and feeder.

We followed a previously published methodology (Ohlinger et al. 2022a, Couvillon et al. 2023) to determine the effect of the treatment on foraging, exposing each feeder to either an untreated or treated device in the experimental phase. We counted the number of visits each individually marked bee made to the feeder over a 6-h period, from 15:00 to 21:00. This is an active time period for foragers and it corresponds to the time when a consumer is likely to use the device. We recorded a visit if the bee landed, extended her tongue, imbibed, and if 3 min had passed from a previous visit (Ohlinger et al. 2022a). We call this discrete, positive count the **foraging frequency** during the experimental phase.

We deployed 2 Canon Vixia HFR82 video cameras on either side of the glass-walled hive to record waggle dances by marked bees during the 6-h experimental phase. We used a SanDisk Extreme SD card to record the videos and then later uploaded them to Google Team Drive for analysis. Bees returning to the hive from a profitable food source communicate to other bees the distance and direction from the hive to the feeding location through the waggle dance (von Frisch 1967, Couvillon 2012).

We performed video analysis to determine **waggle dance propensity** and **waggle dance frequency**. Waggle dance propensity is the proportion of foragers that made any dance at all, yielding a binomial (yes/no) outcome. Waggle dance frequency measured how often a bee repeats a dance (how many dances a bee will perform) with successive foraging trips as a discrete, positive count.

Bees are more persistent in returning to an empty feeder if a site has previously been highly rewarding (Al Toufailia et al. 2013). Therefore, **persistency** is another behavior that indicates bee perception of resource quality. We measured persistency by placing 2 empty, unscented feeders in the same feeder location the next morning (Day 1). We also returned the Thermacell devices to their position from the previous evening, but the devices were not activated as the goal was to understand next-day effects. We counted visitations of marked bees to the empty feeders from 09:00 until 12:00. The discrete, positive count of visitations was our measure of persistency.

Overall, we designed the experiment to monitor foraging and recruitment of bees visiting one of 2 feeders (treatment versus control). Feeders were equally costly (equidistant) and equally rewarding (equal molarity) to visit. Therefore, any differences in monitored behavior indicate a treatment effect. We considered foraging frequency the primary outcome of the study and waggle dance propensity, waggle dance frequency, and feeder visit persistency were considered secondary outcomes.

### Statistical Analyses

We used R 4.2.3 for all analyses (Team 2023). Data are in graphical form and summarized by treatment and trial phase. We show both means (and 95% CI) and medians (and lower and upper quartiles) in these summaries. We derived differences in treatments from mixed models. We provide point estimates for treatment differences in addition to 2-sided 95% confidence intervals (95% CI) and corresponding *P*-values. We analyzed the bee population according to the intention-to-treat principle. That is, the feeder a bee frequented during the training phase determined the treatment in the analysis. Bees that swapped between feeders were exceedingly rare in all trials, and we therefore omitted a per-protocol analysis (Ohlinger et al. 2022a, Couvillon et al. 2023).

We analyzed counts of foraging frequency and dance frequency during the experimental phase, and persistency feeder visits on the day after exposure using Poisson generalized linear mixed-effect models, considering trial-specific and individual-specific intercepts. The observation-specific random intercepts were important because we detected over-dispersion in model checks. We used the glmer function from the lme4 package to model the data (Bates et al. 2015). We employed Binomial generalized linear mixed-effects (function glmer, package lme4 [Bates et al. 2015]) to model the binomial outcome of dance propensity. We calculated treatment group contrasts and extracted 95% CI using the emmeans package (Lenth 2024).

Because of poor recruitment in some trials, which occurs when conditions fluctuate to disfavor feeder training, we decided to include pre-training bees in the analysis. These bees may have had longer exposure to the feeders before treatment and may therefore be more likely to continue foraging even to an unfavorable feeder. We also decided to use bees that were recruited to feeders after onset of experimental exposure. To capture these differences among bees, we used an observation level random intercept to capture these differences among bees and performed a sensitivity analysis, including an offset to correct for the shorter window the latter group of bees had to accumulate visits to feeders and for performing dances. Results were not affected (see below).

The number of visits during training phase bees marked early in the training phase have longer time to train to the respective feeder and form a tighter commitment (Couvillon et al. 2015). This might lead to more visits during the experimental and persistency phases. Therefore, we added number of visits to feeder during the training phase to statistical models in a sensitivity analysis to control for the individual commitment of a bee, regardless of treatment. This may be important because longer experience with a feeder forms a tighter commitment (Seeley 1995, Couvillon et al. 2015), which may impact outcomes.

Individual identifiers consisted of colored discs with numbers that were glued to the thorax of bees in the pre-training days of each experiment. Depending on color combination of the background and the typed number, some individual number tags were not legible in the later video analysis. We assumed these numbers to be randomly missing in our data sheet and didn’t count unidentified dancers for the main analysis of the dance outcomes.

All data and analyses code for this study are available at https://doi.org/10.7294/27091720.

### Test Product Details

A Thermacell E90 repeller was tested using a 5.5% metofluthrin refill. The repeller contains a heating ring near the top of the device, which generates airborne mosquito repellent. The active ingredient, metofluthrin, is incorporated in a hydrocarbon formula at 5.5% by weight. The formula is contained in an acrylic bottle and is drawn up through a polymeric wick to the wick’s tip, which is surrounded by the heating ring. The heated tip of the wick emits metofluthrin and the other formula ingredients into the air. The heating ring is powered by an internal, rechargeable battery that lasts a period of 9 h until the next charge.

A control formula, containing no active ingredient, and a treatment formula, containing 5.5% metofluthrin, were employed in the study. The containers were labeled as DELTA and GAMMA to be blinded to researchers in the field.

Prior to the initiation of the experimental phase, the devices with formula containers were run for 2 h as part of a pre-burn in a location removed from the test site prior to test initiation. This step insured that fresh formula from the container would be emitted in the active portion of the study.

### Presence and Fate of Metofluthrin

Prior to the start of the bee field experiments, two of us (Thermacell scientists C.B. and B.M.) measured the airborne concentration of metofluthrin during product use in the on-site test plots (see Supplementary Information, Bizon, online for full report). We placed passive dosimeters (320 mm diameter filter paper) at intervals of 1.0 m, and 2.0 m from the device in 3 directions, forming angles of 120° from the device (north, southeast, and southwest) and at 0, 1 m, and 2 m above the ground at each location. They were in place throughout the pretest and removed before the onset of the bee field experiment. These dosimeters captured metofluthrin that would fall to surfaces in the treated area. We also placed active air samplers at each dosimeter location at a height of 1m. The AirChek XR5000 air samplers draw air through an adsorbent tube at 2 L/min. The air samplers measured airborne metofluthrin in the test area. Filter papers and air sampling media were extracted with acetone and subsequently analyzed for the presence of metofluthrin using gas chromatography.

Given low rates of deposition to passive dosimeters, Thermacell decided to conduct further laboratory analysis to determine the particle size emitted from the device. We partnered with researchers to use particle size analysis with other product data to model the behavior of metofluthrin emissions from the E-series device and the behavior of prallethrin emissions from the MR300 device (Couvillon et al. 2023) using computational fluid dynamics (CFD) (see Supplementary information, Pham, online for full report).

Aerodynamic particle sizing technique was used to characterize the particle size distribution emitted from the Thermacell devices. Particle size measurements were taken at 20-s intervals over a period of 20 min. Velocity of the particle plume was measured using particle image velocimetry, an optical measurement technique used to analyze fluid flow patterns, tracking the movement of particles over time.

The movement of the particles was modeled under conditions of 50% relative humidity, a temperature of 25°C, and under calm conditions (wind at 0.16 km/h). Ansys Fluent flow solver, version 2022R1, was used to obtain the movement of air particle behavior in the simulation.

## Results

### Product Emission Characteristics

In the product emission pretest, release rate of the formula, as measured by weight loss of the product refill, was consistent with previous measurements, releasing 73 mg of metofluthrin over a 4-h and 27-min test period.

As expected, the pretest of the control product did not find metofluthrin in the passive dosimeters or the active air samplers. The metofluthrin collected from dosimeters in the treated plot was below the limit of quantitation (0.118 µg/cm^2^) at 3 locations and below the limit of detection (0.039 µg/cm^2^) in the remaining fifteen locations, indicating that very little metofluthrin was falling to surfaces in the study plot. The airborne metofluthrin recovered from the active air samplers in the treated plot ranged from below the limit of quantitation (0.0038 mg/m^3^) at 2 m north of the emitting device to 0.0074 mg/m^3^ at 1 m southwest of the device.

The mean particle size of the emissions from the Thermacell E-series device was 4.43 µm. Particles in the range of 1–10 µm are known to remain suspended in the air for 10 to 100 h (Esmen and Corn 1971). The specific settling time for the particles in the E-series simulation was estimated to be greater than 12 h. But, the actual settling time is likely longer since the evaporation of formula components would continue to reduce particle size in the air. The velocity of the formula particles was approximately 0.15 m/s. The CFD simulation found an upward motion of the particles under static conditions. Increasing wind speed even slightly would increase the horizontal movement of the particles. Only 0.02% of the emitted formula landed on a surface in the simulation and that was all within approximately 0.5 m of the device. The remainder of the particles (99.98%) remained airborne. Results were similar to those found with the MR300 device emitting prallethrin, though the prallethrin formula particles had a slightly larger mean size at 5.58 µm.

### Experimental Phase Visitation Raw Numbers

**Fig. 2.**
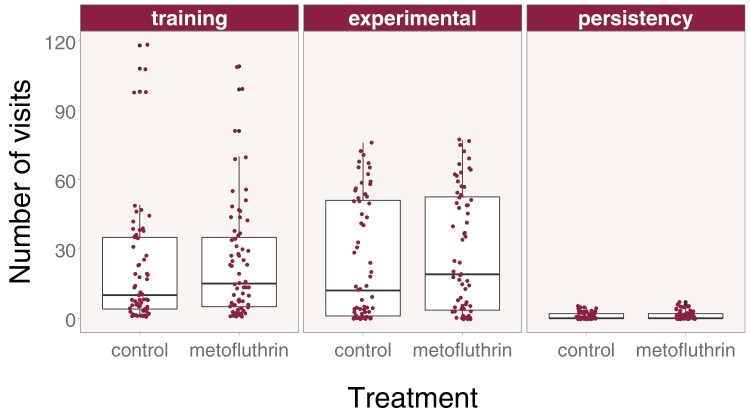
Raw number of visits to the feeders (maroon circles) and boxplots during training. Boxes show interquartile range and the bold line indicates the median. Feeders provided sugar solution during training and experimental phases, but not persistency phase. Bees were only exposed to the treatment during the experimental phase.

We observed little difference in the total number of feeder visits over the 3 stages of the trial. Crude geometric means (95% CI) suggest that bees visited feeders during training 10.90 times (8.62 to 13.77), during the experimental phase 4.45 times (2.58 to 7.66), and during the persistency phase 0.12 times (0.07–0.19). There were no stark differences between treatments for the 3 phases of the experiment (Fig. 2). In particular, our primary outcome, the median number of visitations for bees during the experimental phase, were similar at 12 for control and 19 for metofluthrin treatments.

### Foraging Frequency During the Experimental Phase

**Fig. 3.**
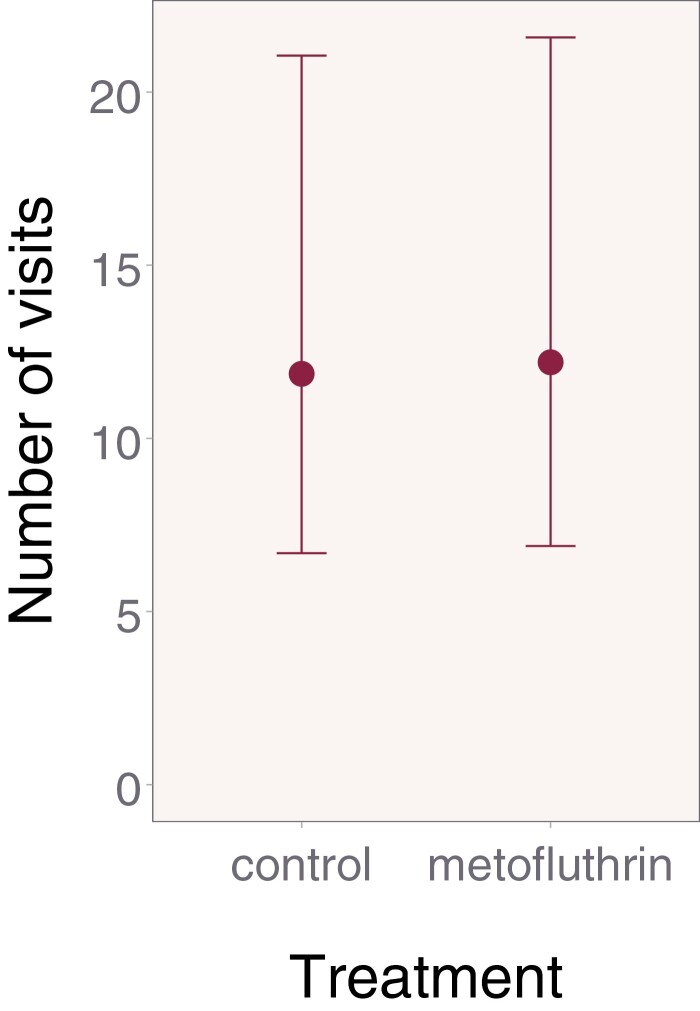
Estimated number of visits to the feeders during the experimental phase. There was no significant difference in foraging frequency, our primary outcome, between our control and treatment bees. Error bars display 95% confidence intervals.

Our mixed-model approach predicted the mean foraging rate was higher in the metofluthrin treatment compared to control bees during the experimental phase, but this was not a significant difference (mean [95% CI]; control: 11.9 [6.7–21.1]; metofluthrin: 12.2 [6.9–21.6]; ratio metofluthrin/control: 1.0 [0.9–1.2]; *z* = 0.41; df = 1; *P* = 0.68; Fig. 3). On average the number of visitations for control bees were only 0.97 times as high compared to visitations for metofluthrin, but the 95% CI indicates the possibility that control visitations could also be higher.

Sensitivity analysis that includes consideration of observation time during the experimental phase also indicates no treatment effect (χ^2^ = 3.24; df = 1; *P* = 0.07). Considering the potential confounding effect of visits during the training phase (see above), we nonetheless find that treatment was not a significant predictor of visits over the experimental phase (χ^2^ = 2.89; df = 1; *P* = 0.09).

Additionally, we carried out a Wilcoxon rank sum test as an additional sensitivity analysis, ignoring the potential correlation within hives as Figure 2 suggests that the Poisson model we pre-specified in the protocol might not be appropriate to the analysis of the primary outcome of experimental phase feeder visits. This test also showed no difference in foraging between control and metofluthrin treatment (*W* = 1915.5, *P* = 0.23).

### Waggle Dance Frequency and Waggle Dance Propensity During the Treatment Phase

The raw counts (Table 1), ignoring potential among hive differences in treatment effects, of dancing and non-dancing bees in the 2 treatments suggest no significant difference in the propensity to dance (χ^2^ = 0.5; df = 1; *P* = 0.49). Additionally, we found by analysis in a generalized mixed-model framework that the propensity to perform waggle dances was strongly dependent on the length of time that bees participated in the experiment (*P* < 0.001; result not shown), regardless of treatment. Once we adjusted for the training effect, we observed no difference in waggle dance propensity between treatments (probability to dance (95% CI): control: 0.6 (0.3–0.9); metofluthrin: 0.6 (0.3–0.9); OR metofluthrin/control (95% CI): 1.0 (0.4–2.6); χ^2^ = 0.0061; df = 1; *P* = 0.94). The lack of significance in waggle dance propensity during the experimental stage was a secondary outcome.

**Table 1.** Number of honey bees that performed a waggle dance (dance propensity, one of our secondary outcomes) in each treatment. There was no significant difference in dance propensity between our treatment and control bees. This was one of our secondary outcomes.

Treatment	Danced	
	No	Yes
Metofluthrin	31	36
Control	35	30

**Fig. 4.**
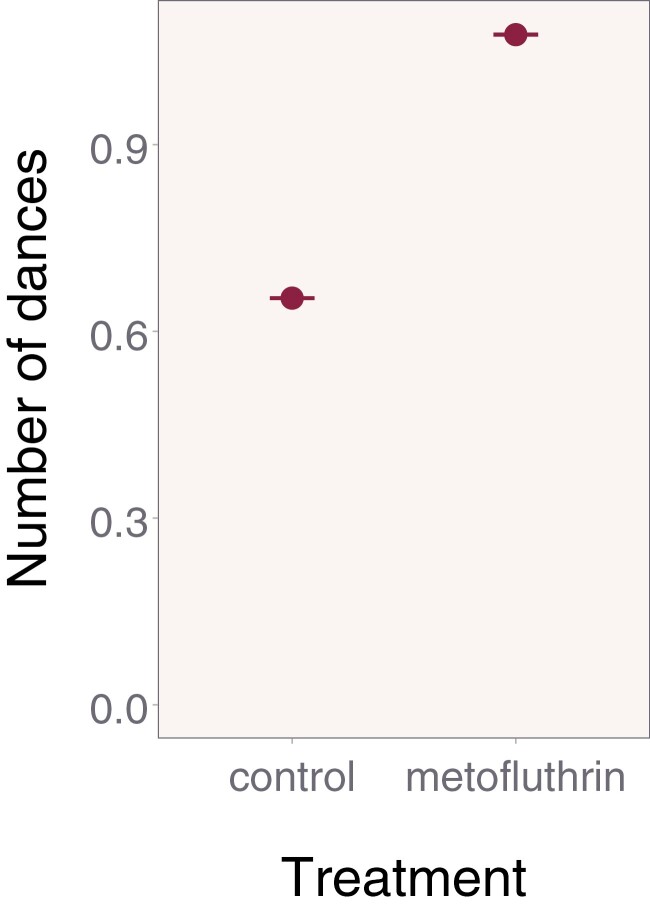
Estimated number of dances during the 6-hour experimental phase. There was a significant difference in dance frequency, one of our secondary outcomes, between our control and treatment bees. Error bars display 95% confidence intervals.

Once a bee began dancing, the number of dances performed during the experimental phase was higher for the metofluthrin treatment compared to the control (Fig. 4). This effect was significant (mean number of dances [95% CI]: control: 0.65 [0.65–0.66]; metofluthrin: 1.08 [1.08–1.08]; OR metofluthrin/control [95% CI]: 1.65 [1.65–1.65]; χ^2^ = 8.01; df = 1; *P* = 0.005). This significant effect in waggle dance frequency during the experimental phase was another secondary outcome.

### Feeder Persistency After the Experimental Phase

**Fig. 5.**
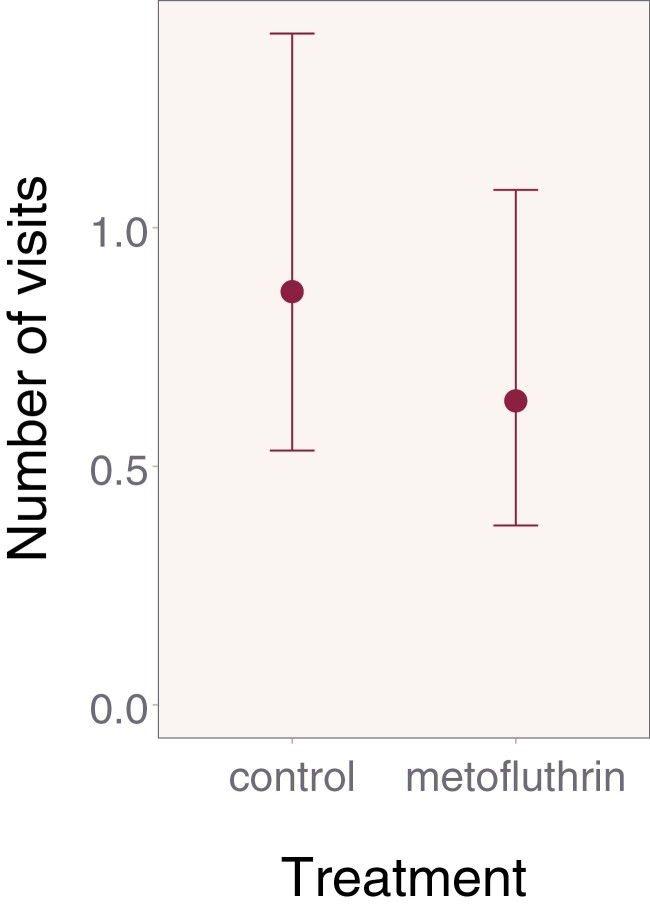
Estimated number of visits to the feeders during the persistency phase. There was no significant difference in persistency, one of our secondary outcomes, between our control and treatment bees. Error bars display 95% confidence intervals.

The mean foraging rate as predicted by the mixed-model approach was numerically lower in metofluthrin compared to control during the persistency phase, but again, non-significantly (mean [95% CI]; control: 0.9 [0.5–1.4]; metofluthrin: 0.6 [0.4–1.1]; ratio metofluthrin/control: 0.7 [0.5–1.2]; *z* = −1.33; df = 1; *P* = 0.18; Fig. 5). The non-significance in feeder visitation during the persistency stage was a secondary outcome.

## Discussion

We did not observe any significant differences in foraging frequency, waggle dance propensity, or persistency in bees collecting sucrose solution next to an #90 Rechargeable Mosquito Repeller device with active ingredient compared to bees collecting next to a device without active ingredient. However, we did see a significant effect on one of our secondary measure’s recruitment behavior (waggle dance frequency). It is worth noting that it is unusual that we see an effect of treatment for a recruitment behavior (waggle dance frequency) when we did not see one with foraging behavior (foraging frequency), as the two are usually in tight concordance. One important consideration is the small sample size for our waggle dance frequency analysis, which was driven by the lower overall recruitment that we experienced here compared to previous studies (Ohlinger et al. 2022a, Couvillon et al. 2023). For example, the Y axis units in Fig. 4 are less than 1, whereas previous studies are magnitudes different. The smaller sample size reduces the power and increases the chances of spurious results, as the study is powered for the primary endpoint only. Overall, and in summary, with the non- significant difference in foraging frequency, as our primary outcome, and in all of our other secondary outcomes, we still conclude that honey bee foraging and recruitment were not adversely affected by the device.

One potential explanation for the difference in sample size between this experiment and previous studies could relate to the extended experimental phase, occurring later in the evening (15:00–21:00) than previous work, which concluded no later than 16:30 (Ohlinger et al. 2022a) or 20:00 (Couvillon et al. 2023). This caused bees to keep foraging, but they generally stopped dancing with the approach of nightfall. We note that although persistency did not differ between treatment and control, the treatment bees actually tended to be less persistent, although not significantly so, which is in the opposite direction of the waggle dance frequency. These behaviors typically scale together (Seeley 1995, Al Toufailia et al. 2013, Couvillon et al. 2015), which further suggests that the waggle dance frequency data might be spurious.

Honey bees are exceedingly responsive to reward, especially molarity, and vary their foraging and recruitment behaviors in accordance with how lucrative a resource may be (von Frisch 1967, Seeley 1989, 1995, Couvillon 2012, Couvillon et al. 2014, 2015). The control and treatment feeders contained identical 1M sucrose solutions and were equidistant from the hive. So, cost and profitability were equivalent, which means that differences in behavior would be solely the result of the metofluthrin emitted from the Thermacell device. Honey bees are also more likely to perform the waggle dance and will dance more often if the forager is visiting a very lucrative resource (Seeley 1995, Couvillon 2012). The same forager will consistently return to a resource, even after it becomes unprofitable (Al Toufailia et al. 2013). The lack of adverse effects on honey bee behavior suggests that bees were either unaffected or not affected in ways reflected by the measured behaviors.

Why would emissions that are demonstrated to affect another insect’s behavior, mosquitoes, not affect honey bee behavior? A similar result was observed with another Thermacell emitting device (MR 300) that produced tiny, airborne particles of a different active ingredient, prallethrin, and yet did not seem to affect honey bee foraging, recruitment or persistency (Couvillon et al. 2023). Other studies have demonstrated that honey bees do experience lethal and sublethal effects due to pyrethroid exposure (Ingram et al. 2015, Johnson 2015). This suggests that heating pyrethroids to form airborne, micron-sized particles, reduces or eliminates the negative effects of these ingredients on honey bees. One possibility for the difference versus mosquitoes is that the active chemical concentration produced by the Thermacell devices is insufficient to affect the more robust honey bees. But this would seem less likely if the reaction of mosquitoes to the airborne chemical is sensory. Mosquitoes and honey bees both have very sensitive olfactory systems used in host or flower-finding and, in the case of honey bees, in communication.

Instead, it may be a difference in how flight towards a food source is directed in the 2 insects. Mosquitoes encountering increasing airborne concentrations of metofluthrin are searching initially via olfactory cues, following traces of a carbon dioxide plume emanating from a host (Cardé 2015). Metofluthrin has been shown to act in mosquitoes by either causing directed movement away from the ingredient, repellency (Lucas et al. 2007), or by causing disorientation (Rapley et al. 2009). In both cases, repellency or disorientation, the normal response to carbon dioxide or other attractants is countered by the presence of metofluthrin. Honey bees visiting a food source are usually either experienced foragers with a memory of the forage (Grüter and Ratnieks 2011, Grüter et al. 2011) or have been recruited to a food source through the waggle dance in the hive and respond to visual cues such as the sun and other objects that help them gauge distance (von Frisch 1967, Couvillon 2012). The presence of airborne metofluthrin might be less likely to influence their travel to a food source if visual cues are primary. Another possibility could be that the scent or the taste of nectar has triggered a memory of the route to the feeder (Grüter and Ratnieks 2011). It is also possible that honey bee flight patterns to a resource differ from those of mosquitoes in a way that reduces their encounter with airborne metofluthrin. Honey bees recruited by waggle dances follow a direct, approach to the vicinity of a feeder (Riley et al. 2005). Mosquitoes, on the other hand, follow a surge-cast approach, involving an upwind surge in response to carbon dioxide and then side-to-side casting in search of another filament of CO_2_ (Dekker and Cardé 2011). It seems possible that such casting behavior would result in greater exposure to airborne pyrethroids, resulting in disorientation or repellency. Modeling indicates that particles from the Thermacell device rise into the air and may be gradually swept upwards as they disperse. Would mosquitoes be more likely to encounter greater amounts of metofluthrin as they follow wisps of carbon dioxide, which may be similarly swept upwards from their source? Is it possible that bees fly under the higher concentrations of metofluthrin? Furthermore, the finding that metofluthrin dispensed by spatial repellent devices remains airborne as it diffuses in the air suggests that honey bees would encounter little, if any, metofluthrin on the surfaces of nectar and pollen gathering sources. As a result, honey bees would not be dissuaded from landing, walking on, and ultimately collecting from these surfaces.

The analysis we pre-specified in the protocol for the primary outcome was a Poisson mixed model. It appears that we bumped up against an upper limit of visitations during the experimental phase that was set to 6 h, and the data appear not to be Poisson distributed. This might be due to the feeders being too rewarding, and future studies might consider a lower molarity of sucrose solution, with once again keeping molarity the same between treatment and control. Despite this limitation, a non-parametric sensitivity analysis suggests that our inference of a non-effect of treatment is valid.

Our findings here are consistent with the previous study on the effect of the pyrethroid prallethrin emitted from a heated surface in that we found no honey bee morality and no adverse effect on honey bee foraging and recruitment (Couvillon et al. 2023). Based on the supplementary studies of pyrethroid emissions and their behavior in the air, it appears that these results are driven by the size of the emitted particles, their tendency to remain airborne and perhaps their airborne concentration, rather than properties of the insecticides themselves. Further study of the interaction of mosquito and honey bee sensory apparatus with these pyrethroids would be useful to determine whether these organisms react differently to airborne pyrethroids, or whether the insects have different levels of sensitivity to these chemicals. While limited to honey bees over a relatively short exposure, our research suggests the possibility that similar spatial repellent products may be used without disrupting the activity of related insect pollinators in the field.

## Supplementary Material

ieae103_suppl_Supplementary_Material
