# Supplementary material for: Airborne metofluthrin, a pyrethroid repellent, does not impact foraging honey bees
Source: J Insect Sci. 2024 Oct 23;24(5):7. doi: 10.1093/jisesa/ieae103 (PMC11497606; doi:10.1093/jisesa/ieae103)
Supplement: ieae103_suppl_Supplementary_Material [file ieae103_suppl_supplementary_material.zip › Anon_SI Bizon Thermacell Test Data Summary of TRI-CHM-016.pdf]

Technical Memorandum

To: [redacted]

Date: 10/31/2023

Subject: **Residual Metofluthrin Examination of Spatial Repellents to Supplement Pollinator Study**

Project #: **TRI-CHM-016**

BACKGROUND

The reachable line of Thermacell insect repellers is well-known to have strong repellency to mosquitoes in a variety of conditions. To better understand the performance of this product, it was determined that it would be beneficial to grow the understanding of any potential effects on pollinators. This testing would be performed by the Virginia Tech department of Entomology. Thermacell's science and research division would need to perform a deposition study to confirm that the devices were functioning appropriately.

METHOD

A Thermacell E-55 rechargable repeller with a full repellent refill was placed in the identical testing location as the corresponding pollinator study. Six data collection stations were placed surrounding the repeller as seen in Figure 1. Each test station was equipped with three pieces of filter paper, each secured by a ring stand, to act as passive dosemeters. These filter papers were at the following heights: 0 meters (ground level), 1 meter, and 2 meters. Each test station also featured an AirChek XR5000 personal air sampling pump equipped with flexible tubing to connect the pump to a OVS-2 (OSHA Versatile Sampler) adsorbent tube obtained from Supelco (ORBO 49-P). These pumps were calibrated to run at 2L/min using a Bios Defender Calibrator. This setup was replicated for a second test system in a nearby area that was far enough away to avoid any potential cross contamination of the systems. Thermacell's rechargable line of products typically utilize a formulation containing 5.5% metofluthrin. A rechargable repeller was loaded with one of these liquid refills, while a control refill containing no metofluthrin whatsoever was loaded into an identical rechargable repeller in a second test system.

Figure 1. Testing Configuration and Test Station Illustration.

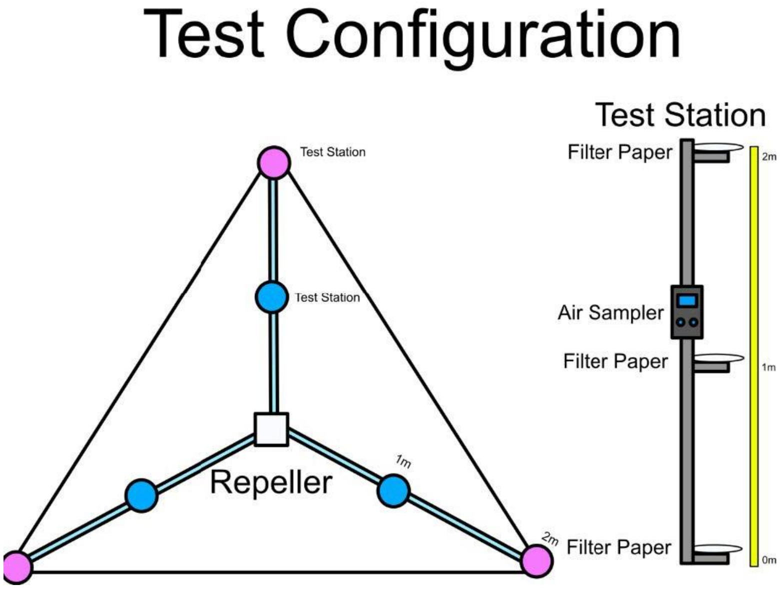

The AirChek XR5000 air sampling pumps were calibrated at 2.0L/min, and their absorbent tubes (ORBO 49-P, Supelco, Inc.) were uncapped and prepared for use. All air sampling pumps and test systems repellers were then activated. The system was allowed to run for four hours. Once the experiment concluded, the air sampling pumps were turned off, their media collected, and each filter paper was collected, placed in a 50ml centrifuge tube, and sealed. All samples were then transported to the Thermacell laboratory, where they were kept at 5°C until processing. The used mats were kept and reweighed post testing to determine the overall material lost.

The filter papers were then extracted in their centrifuge tubes with acetone. The filter paper was extracted with three washes of acetone (1 x 40 mL, 2 x 30mL) and 10 minutes of sonication per wash. The liquid extract was transferred into a 100mL volumetric flask between washes. The 100 mL volumetric flask was topped up to the mark with acetone and mixed by inversion. Then, the extracted sample was diluted 1:1 with internal standard and then analyzed by GC-MS using a previously validated method.

All collected air sampling media was rinsed three times with 10ml of acetone. All the washes were transferred into a 100ml volumetric flask between washes. The volumetric flask was then topped up to the 100ml mark with acetone and mixed by inversion. The sample was then extracted and diluted 1:1 with internal standard and analyzed via GC-MS using a previously validated method. This method was constructed by the dilution of the active ingredient, Metofluthrin (97.0% analytical standard) to known concentrations with internal standard (dibutyl Phthalate) to construct a standard curve, limit of detection, limit of quantification, and the resulting spike recovery. Quantitation is achieved by comparison of the field samples against this standard curve.

RESULTS

The concentration of each liquid refill was determined via GC-MS prior to the experiment. The formulation containing metofluthrin was labeled “Delta” to prevent cross contamination with the control refills.

Table1. Metofluthrin concentration in test

Concentration of Metofluthrin (w/w%)	
“Delta” (Metofluthrin refill)	Control Group
5.51	0.00

Once the experiment was complete, the concentration of the active ingredient, Metofluthrin, was determined via GC-MS for the filter papers, and air sampling media. A spike recovery was completed for each method to observe accuracy. The limit of detection and limit of quantification for each method was calculated.

Table 2. “Delta” Metofluthrin Refill Release Information

Overall material released (mg)	Approximate Metofluthrin Released (mg)	Time Released	Device Temperature (°C)
1325	73	4 hours, 27 mins	173

Table 3. Metofluthrin Concentration on Filter Papers

Distance From Emitter	Height	Concentration of Metofluthrin (µg/cm^2) - Metofluthrin refill	Concentration of Metofluthrin (µg/cm^2) - Control refill
1.0m (North)	Ground level	<LOD	<LOD
	1m	<LOQ	<LOD
	2m	<LOD	<LOD
1.0m (South-East)	Ground level	<LOQ	<LOD
	1m	<LOQ	<LOD
	2m	<LOD	<LOD
1.0m (South-West)	Ground level	<LOD	<LOD
	1m	<LOD	<LOD
	2m	<LOD	<LOD
2.0m (North)	Ground level	<LOD	<LOD
	1m	<LOD	<LOD
	2m	<LOD	<LOD
2.0m (South East)	Ground level	<LOD	<LOD
	1m	<LOD	<LOD
	2m	<LOD	<LOD
2.0m (South-West)	Ground level	<LOD	<LOD
	1m	<LOD	<LOD
	2m	<LOD	<LOD

Table 4. Atmospheric Concentration of Metofluthrin in test. Pump rate of approximately for each pump

Distance from Emitter	Concentration of Metofluthrin Observed (mg/m^3) - Metofluthrin Refill	Concentration of Metofluthrin Observed (mg/m^3) - Control Refill
1.0m (North)	0.0062	<LOD
1.0m (South-East)	0.0069	<LOD
1.0m (South-West)	0.0074	<LOD
2.0m (North)	<LOQ	<LOD
2.0m (South-East)	0.0043	<LOD
2.0m (South-West)	0.0041	<LOD

Table 5. Limits of Detection and Quantification for Test System

Limit of Detection for GC/MS (µg/mL)	Limit of Quantification for GC/MS (µg/mL
0.159	0.423
Limit of Detection for Filter Paper ($µg/cm^2$)	Limit of Quantification for Filter Paper ($µg/cm^2$)
0.039	0.118
Limit of Detection for Sample Tube (mg/m^3)	Limit of Quantification for Sample Tube (mg/m^3)
0.0022	0.0038

Table 6. Filter Paper Recovery of Spike in Various Conditions.

Location	Laboratory Spike	Field Spike	Field Spike
Dry Time	10 minutes	10 minutes	4 hours, 27 minutes
Replicate	Metofluthrin Spike Recovery (%)		
A	98.2	95.8	84.1
B	97.5	93.9	82.2
C	97.3	93.4	86.7
D	95.6	94.0	88
E	98.8	96.7	85.5
**Mean**	97.5	94.76	85.3
**Stdev**	1.21	1.42	2.25
**% RSD**	1.24	1.49	2.64

Table 6. OVS Air Sampling Tubes Spike Recovery.

Replicate	Metofluthrin Spike Recovery (%)
A	98.9
B	99.5
C	99.2
D	99.4
E	99.6
**Mean**	99.32
**Stdev**	0.28
**% RSD**	0.28

CONCLUSION

The deposition of metofluthrin was observed for two test systems. The first test system utilized a typical metofluthrin containing liquid refill, while the second test system used a control formulation that had no traces of metofluthrin. The system utilizing the metofluthrin containing formulation emitted metofluthrin at a level consistent with previous experiments and shows that the device and its liquid refill functioned properly in this study. The control formulation showed no detectable concentrations of metofluthrin in any of the material tested.
