# Supplementary material for: Airborne metofluthrin, a pyrethroid repellent, does not impact foraging honey bees
Source: J Insect Sci. 2024 Oct 23;24(5):7. doi: 10.1093/jisesa/ieae103 (PMC11497606; doi:10.1093/jisesa/ieae103)
Supplement: ieae103_suppl_Supplementary_Material [file ieae103_suppl_supplementary_material.zip › SI Pham Technical report - particle emission from Thermacell devices Final.pdf]

Fate and Transport of Particles Released from Thermacell Mosquito Repellers: A Perspective from Computational Fluid Dynamics Simulation

Ngoc Pham, Ph.D.

Advanced CAE, Science & Innovation Incubator, SC Johnson

Introduction

Insect-borne diseases pose a significant threat to human health, with millions of people affected worldwide each year. The use of insect repellents has long been recognized as an effective strategy to mitigate the risk of insect bites and subsequent disease transmission. However, understanding how Spatial Repellent Devices (SRD) work or evaluating the repellent efficacy has traditionally relied on empirical testing, which can be time-consuming, expensive, and ethically challenging. In recent years, computational fluid dynamics (CFD) simulation has become a valuable tool for investigating the efficacy of insect repellents in a controlled and efficient manner. Numerous studies have already embraced the use of CFD simulation in their research on insect repellents. For instance, Stevenson et al.[1] highlighted the need of spatial repellents as a supplement to existing vector control tools for malaria control in Zambia, as they are threatened by mosquito resistance to insecticides and changes in mosquito behavior. They conducted a semi-field evaluation of a novel SRD, which emanates metofluthrin without the need for external power or heat. It was found that the presence of the SRD resulted in greater indoor catches of mosquitoes, which may be explained by the excito-repellency activity of metofluthrin. CFD simulations were also employed to predict the concentration of metofluthrin inside and outside the hut where the SRDs were deployed. Bernier et al.[2] combined entomological experiments with CFD simulations to assess the effectiveness of a novel device for controlled release of spatial repellents against mosquitoes. They tested SRDs with different concentrations of metofluthrin against Anopheles mosquitoes in laboratory, semi-field, and outdoor settings, and correlated the experimental results with simulation results to develop a functional model that links mosquito mortality with the estimated spatial concentration of the repellent. Siegel et al.[3] researched tick bite prevention by using spatial repellents, which deter ticks from landing on a treated surface by interfering with their odorant receptors. Their experiments involved exposing ticks to transfluthrin and metofluthrin using SRDs and observing their behavior such as climbing activity, detachment from climbing sticks, and pseudo-questing duration to evaluate the effects of the repellents. CFD simulations were also used to understand the distribution and movement of the repellent formulations (transfluthrin and metofluthrin) in the test chamber. From a safety perspective, Wang et al.[4] studied the emission characteristics and health risks associated with burning mosquito-repellent incense. They quantitatively characterized the major gaseous and particulate pollutants emitted from

different types of mosquito-repellent incense, including solid, electric liquid, and electric mat types. Dispersion and concentration distribution of pollutants in a room with windows opened and closed were also evaluated by using CFD simulations. The simulations helped assess the effectiveness of ventilation in reducing pollutant concentrations and provided insights into the potential adverse health effects of exposure to these pollutants.

In this study, we used CFD simulation to provide a qualitative and quantitative understanding of the fate and transport of particles emitted from Thermacell MR300 Portable Mosquito Repeller and Thermacell E-series Rechargeable Mosquito Repeller (referred to as paper mat and rechargeable devices, respectively) when used in an outdoor environment. The paper mat device is designed to effectively manage mosquito populations in outdoor spaces. This product consists of a cellulose mat infused with a concentrated liquid containing ETOC, released into the air by heating the cellulose mat. The radius device, on the other hand, uses a heated wick to aerosolize the mosquito repellent liquid.

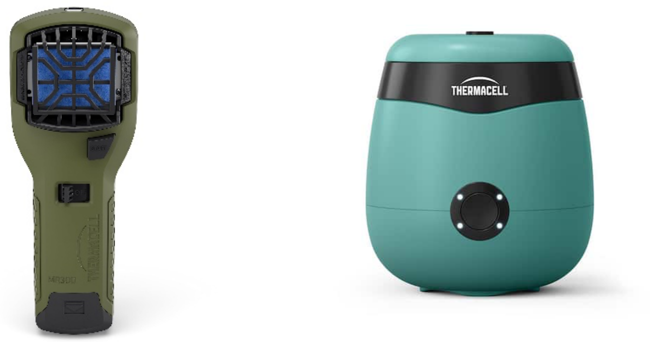

Figure 1. Thermacell MR300 Portable Mosquito Repeller (the paper mat device; left) and Thermacell E-series Rechargeable Mosquito Repeller (the radius device; right)

<u>Particle emission characterization and simulation setup</u>

- *Particle size distribution*

The characterization of particle size emitted from both the paper mat and the radius device was conducted using aerodynamic particle sizing technique. To accurately capture the dynamic changes in particle sizes that occur during the warming up period of the devices, the size measurement was meticulously replicated at 20-second intervals. This approach ensured that the evolving nature of the particles was adequately accounted for. The measurement process was concluded after 20 minutes, at which stable emission temperature had been reached.

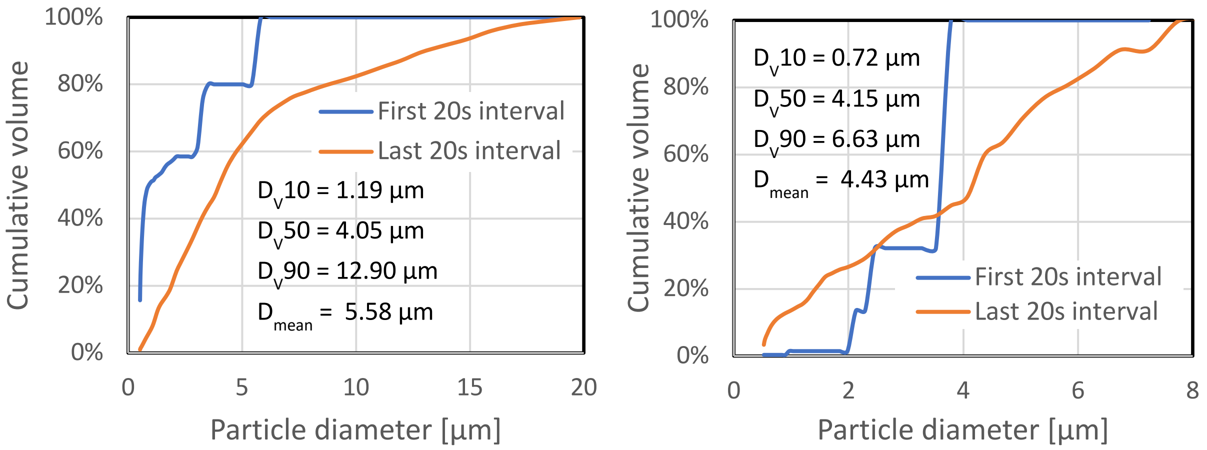

Figure 2. Volume-based particle size distribution of particles, emitted from paper mat device (left) and radius device (right), at the first and last measurement

Figure 2 provides a visual representation of the particle size distribution obtained from two devices. The focus is primarily on the first and last replication in the measurement series. These particular replications were chosen as they offer a clear insight into the evolution of particle sizes during the ramp up period of the emission temperature. It is clear that the particles have changed significantly, spanning a wider range of sizes after 20 minutes. Consequently, the size distribution observed in the last replication is adopted in the simulation model, as it demonstrates a considerably stable state. This choice ensures that the simulation accurately reflects the particle behavior after being released.

Comparing the Dv10, Dv50, and D_{mean} values (embedded in the plots) of the particles of the last replication from both devices reveals their similarity. However, the Dv90 value of the particles emitted from the paper mat device is approximately twice as large as that of the particles from the rechargeable device. Despite the difference, it is noteworthy that particles from both devices are significantly small and are expected to remain suspended in the air for extended periods of time. Particles in the range of 1-10um remain suspended in air on the order of 10-100 hours.[5]

- *Velocity of particle plumes*

Velocity of the particle plumes emitting from the wick of the rechargeable device and from the heated mat of the paper mat device were measured by using the Particle Image Velocimetry (PIV). This is an optical measurement technique used to analyze fluid flow patterns. It involves capturing images of tiny particles suspended in the fluid and then tracking their movement over time. By comparing the positions of particles between consecutive images, the velocity vectors of the fluid can be determined.

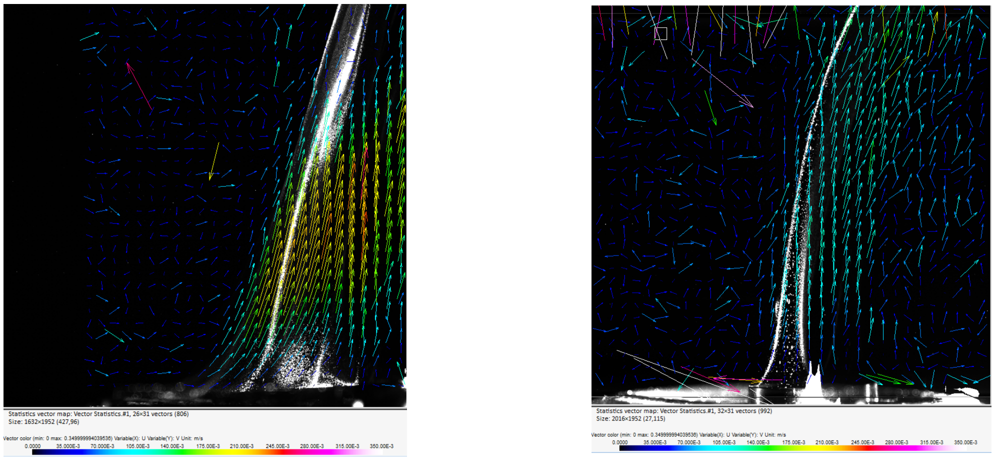

Figure 3. Velocity vectors, colored by velocity magnitude, of particle emission from paper mat device (left) and radius device (right)

Figure 3 illustrates the velocity vectors of the particle plumes emitted by the two devices in a stable emission state. Each figure within Figure 3 represents a statistical average derived from a set of 300 velocity vectors, which were captured over a 20-second emission period. A comparison of the velocity vectors reveals that the particles emitted from the paper mat device exhibit noticeably higher velocities compared to those emitted from the radius device. On average, the particle velocity from the paper mat device is calculated to be approximately 0.2 m/s, while that from the rechargeable device is approximately 0.15 m/s. These average particle velocities were accordingly adopted as initial particle velocities in the simulation models. In addition to the particle emissions, to account for natural convection caused by heating the cellulose mat and heating the wick, the simulation models also assumed hot air currents rising from the heating plate of the paper mat device and the wick of the radius device. The hot air current from each device was assigned the same velocity as the initial velocity of the particles emitting from that device.

- *Temperature of particle plume*

In the simulation models, accurately considering the emission temperature of particle plumes is of utmost importance due to their generation through heating mechanisms. As the transport and thermodynamic properties of the particles are temperature-dependent, understanding the emission temperature is crucial for accurate predictions of the particle behavior after their release. In the simulation models, the emission temperature of particles is assumed to be the air temperature in close proximity to the heat sources (i.e., the wick and the heating plate).

When considering the rechargeable device, it is reasonable to assume that the air in close proximity to the wick maintains a constant temperature due to the small cross-sectional area of the wick. Through our measurements, we determined this temperature to be 72 ^{o}C. However, in the case of the paper mat device, the larger size of the heating plate introduces spatial dependence in the temperature of the air layer near the plate surface. To account for this spatial dependence, we measured the temperature at four points near the four corners of the plate and used nonlinear interpolation to obtain the temperature distribution (see figure 4). This approach allows for a more accurate representation of the temperature profile in the simulation models for the paper mat device.

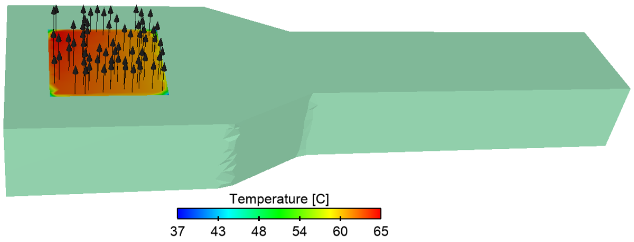

Figure 4. Emission temperature distribution, obtained by nonlinear interpolation, of the air layer close to the heating plate surface of the paper mat device. This temperature distribution was adopted in simulating the paper mat device.

- *Simulation domain and conditions*

The simulation conditions are based on real life conditions which include the following:

Simulation domain: land area with a diameter of 28 meters and a height of 15 meters from the ground. At the center of this land, there is a table that stands 0.9 meters tall (see figure 5). The devices considered are placed on top of this table. The domain has an initial relative humidity of 50 % and temperature of 25 ^{o}C.

Wind at 0.04 m/s, 25 ^{o}C, and 50 % of relative humidity is driven into the domain through a wind tunnel and exits through the domain boundary. According to the Beaufort wind scale, wind speed of 0.04 m/s is described as calm conditions, which smoke rises vertically and sea like a mirror. This is essentially a static air condition, which rarely exists in nature but was adopted for the model to assess worst case conditions for deposition from the products. Increasing rates of airflow of airflow would result in increased particle evaporation rates, thus smaller particle sizes, greater horizontal flow, and longer particle residence time in the air.

In the simulation models, particles emitted from the simulated devices contain ingredients that match those disclosed in the devices' safety data sheets. A 30-minute, stable emission is considered for the paper mat device, while that of the radius device is 15 minutes.

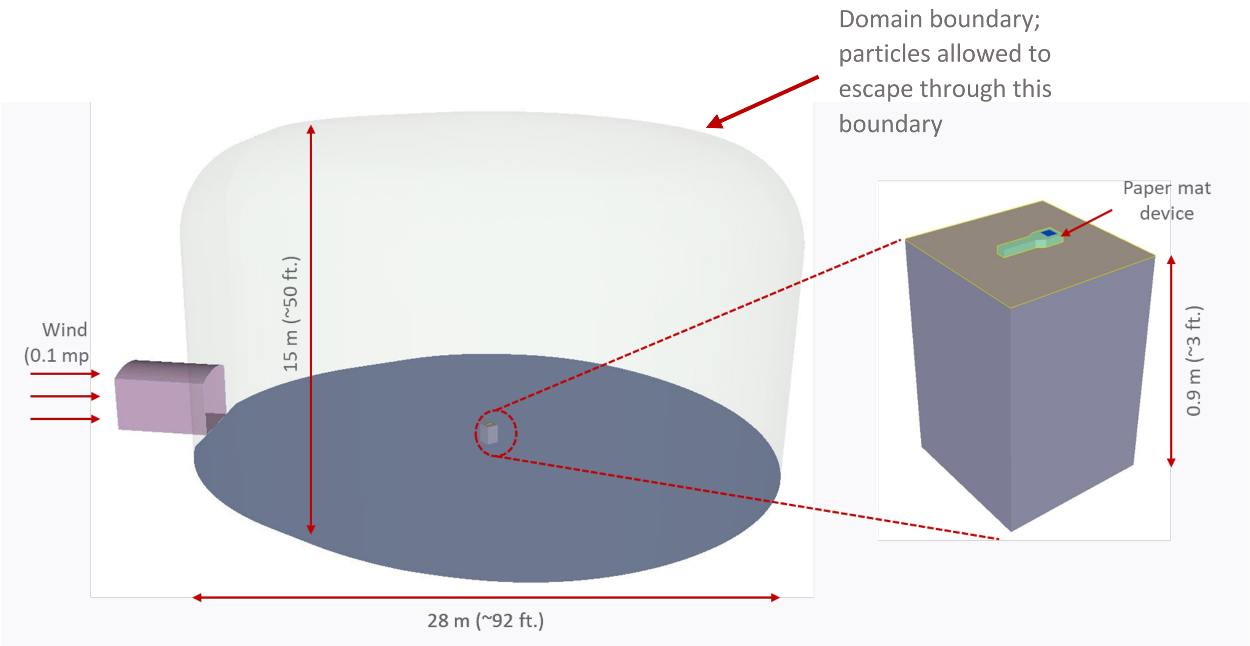

Figure 5. Setup of simulation domain with the paper mat device on the table

Numerical considerations

In this work, Ansys Fluent flow solver, version 2022R1 is employed to obtain the movement of air and particle behavior in the simulation domain by solving the Navier – Stokes equation coupled with the convective, diffusive mass transfer over particle surfaces. Detailed discretization of the Navier – Stokes equation and other equations involved on the computational grid are not presented herein, but the formulation of the convective – diffusive mass transfer model for particle evaporation is briefly described hereafter.[6]

Mass of species i in a particle evaporating during time t is governed by gradient diffusion from the surface exposed to the gas phase, and can be calculated as:

$$m(t) = \dot{N}_\iota A_p M_{w,i} t \qquad (1)$$

where

A_p: surface area of the droplet

$M_{w,\,i}$: molecular weight of species i

t: time

$\dot{N}_\iota$: molar flux of surface evaporation

$\dot{N}_\iota$ can be known if mass transfer coefficient and concentration gradient of species i at the particle surface are known:

$$\dot{N}_i = k_m(C_{i,\,s} - C_{i,\infty}) \qquad (2)$$

where

$C_{i,s} = \frac{p_{sat(T_p)}}{RT_p}$: concentration of species i at the particle surface

$C_{i,s} = x_i \frac{p}{RT_\infty}$: concentration of species i in air

k_m: mass transfer coefficient

$p_{sat\,(T_p)}$: saturated vapor pressure of species i at particle temperature T_p

p: ambient pressure

x_i: mole fraction of species i in the particle

R: gas constant

T_∞: ambient air temperature

Mass transfer coefficient, k_m depends on molecular diffusion of species i and convection in the simulation domain as below:

$$\frac{k_m d_p}{D_{12}} = 2 + 0.6 Re_d^{1/2} Sc^{1/3} \quad (3)$$

where

d_p: particle diameter

D_{12}: diffusivity of species i vapor in air

$Sc = \nu/D_{12}$: Schmidt number

$Re_d = ud_p/\nu$: Reynolds number

ν: kinematic viscosity of air

u: velocity of air layer close to the particle surface

Results and discussions

- *Air movement in the domain under the wind impact*

Figure 6 illustrates the air movement within the simulation domain when subjected to the force of the wind and emission of the particles from the devices. The figure reveals the presence of two prominent air streams. The first stream originates from the wind tunnel, where the wind enters the domain, while the second stream is a vertical airflow generated by the devices. However, these streams lack the intensity required to exert a substantial influence on the surrounding air. This is evident from the sluggish air velocity observed at locations situated away from the main streams' direction of movement. Consequently, it is expected that the current, static air conditions are not favorable for the widespread dispersion of particles emitted by the devices throughout the domain.

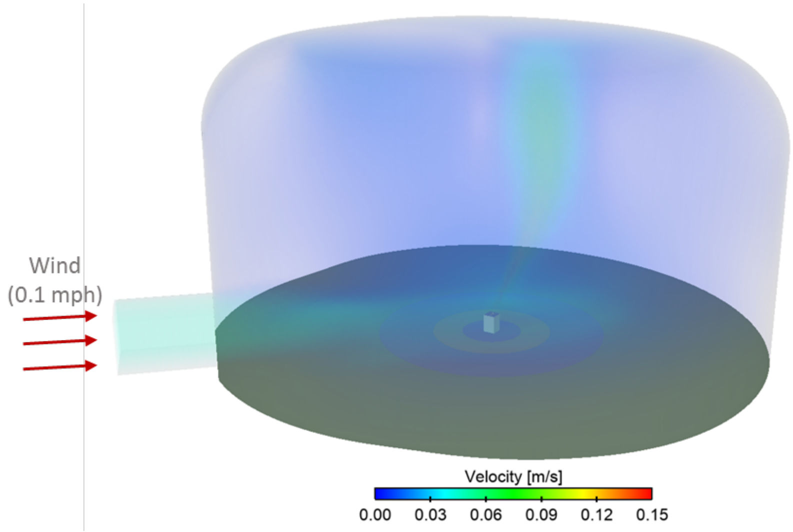

Figure 6. Velocity distribution of air in the studied domain under wind impact and emission of particles

- *Behavior and deposition of particles form the paper mat device*

It is evident from figure 7 that the particles emitted from the paper mat device under static conditions rise upward into the atmosphere. The prevailing wind speed is insufficient to influence their vertical trajectory, indicating that these particles possess the necessary inertia to sustain their upward motion. Furthermore, it is worth noting that certain particles, despite initially descending towards the ground, experience a forceful upward air current generated by the device, causing them to be propelled back into the air. However, not all of them are airborne. Throughout the 30-minute emission period, the cumulative mass of deposited particles amounts to approximately 1.6% of the total formula mass released into the air. It is important to note that this deposited formula mass is exclusively found on the surface of the paper mat that is situated adjacent to the heating plate (see figure 8).

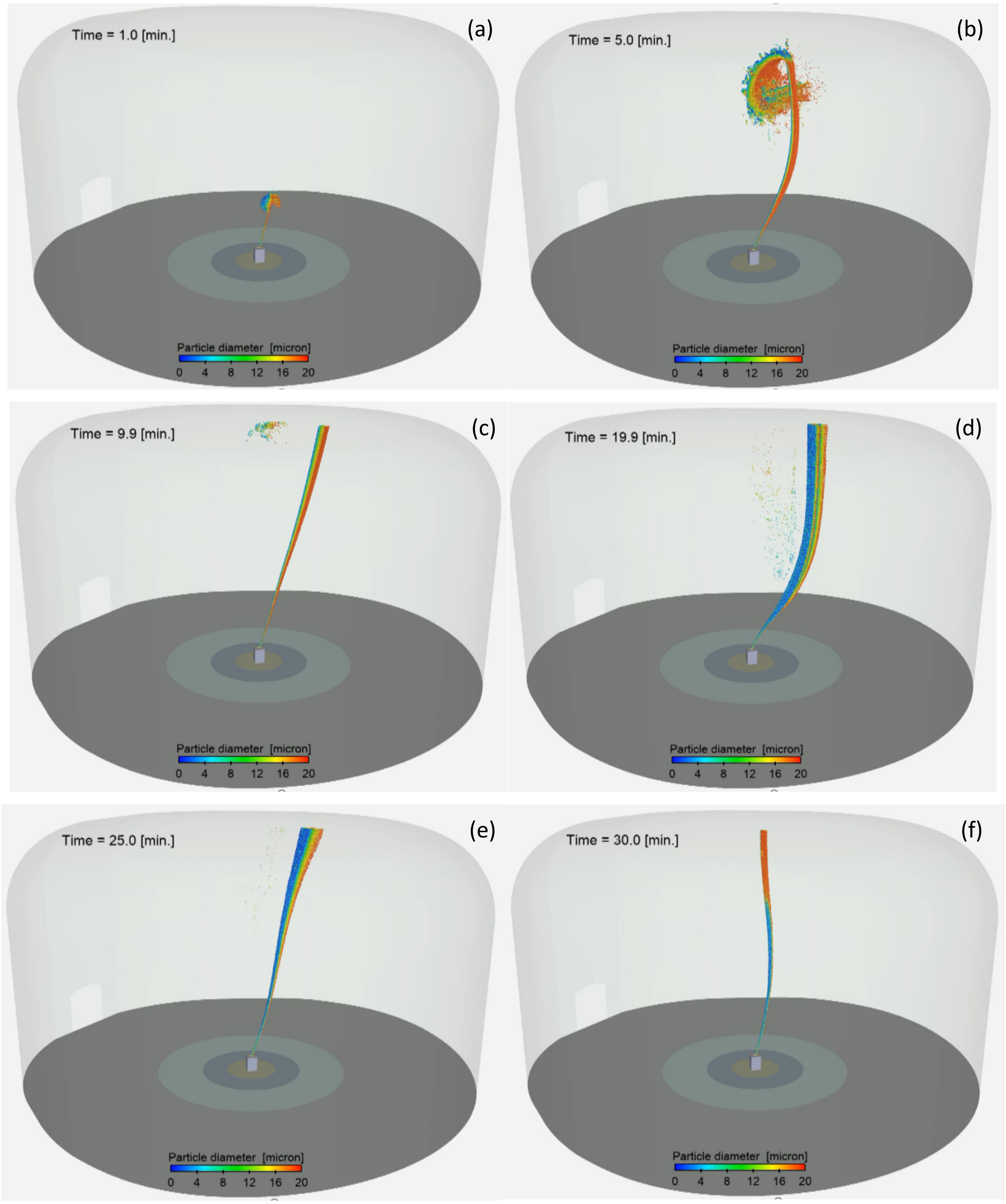

Figure 7. Particle emission from the paper device at (a): 1 minute; (b): 5 minutes; (c) ~10 minutes; (d): ~20 minutes; (e): 25 minutes; (f): 30 minutes. Due to weak wind impact, particles rise right upward without spreading

The deposition of formula on the paper mat surface can be attributed to particles in close proximity to the sides of the heating plate. This observation is linked to the non-uniform air velocity distribution across the plate surface, particularly near the perimeter. In the simulation model, the air velocity in these regions approaches zero, resulting in particles released in those areas lacking sufficient vertical momentum to remain suspended. Consequently, these particles descend and settle onto the nearest surface, which in this case is the paper mat.

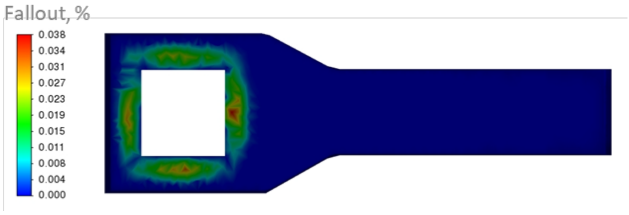

Figure 8. Local deposited mass (fallout) percent on the paper mat surface after 30 minutes of particle emission from the paper mat device. The total deposited mass is 1.6 % of the total formula emitted into air

The consistent finding of particle deposition exclusively on the surface of the paper mat, while being undetectable on the ground and table surface, is in line with the results of a formula deposition study conducted in a large airplane hangar.[7] The main objective of this study was to determine the average concentration of particles deposited on nearby surfaces in a simulated outdoor environment during the use of the paper mat device. The study concluded that particle deposition is undetectable within the 4.5 m circular test area, taking into account the test method's limit of detection, which is 0.000474 µg/cm^2. Details about the study such as test system and procedure can be found in the study’s report.

- *Behavior and deposition of particles form the rechargeable device*

The behavior of particles from the radius device differs significantly from those of the paper mat device. Unlike particles from the paper mat device, particles from the radius device show a stronger dependence on wind impact as they are shifted more to the side boundary (see figure 9). This discrepancy can be attributed to the smaller size of the particles from the radius device, as apparent from their size distribution curves. Smaller particles have less inertia effects, making them more responsive to external forces such as wind. As a result, particles from the the radius device exhibit a wider dispersion pattern compared to those from the paper mat. This is consistent with the more extensive mosquito repellency area produced by these products.

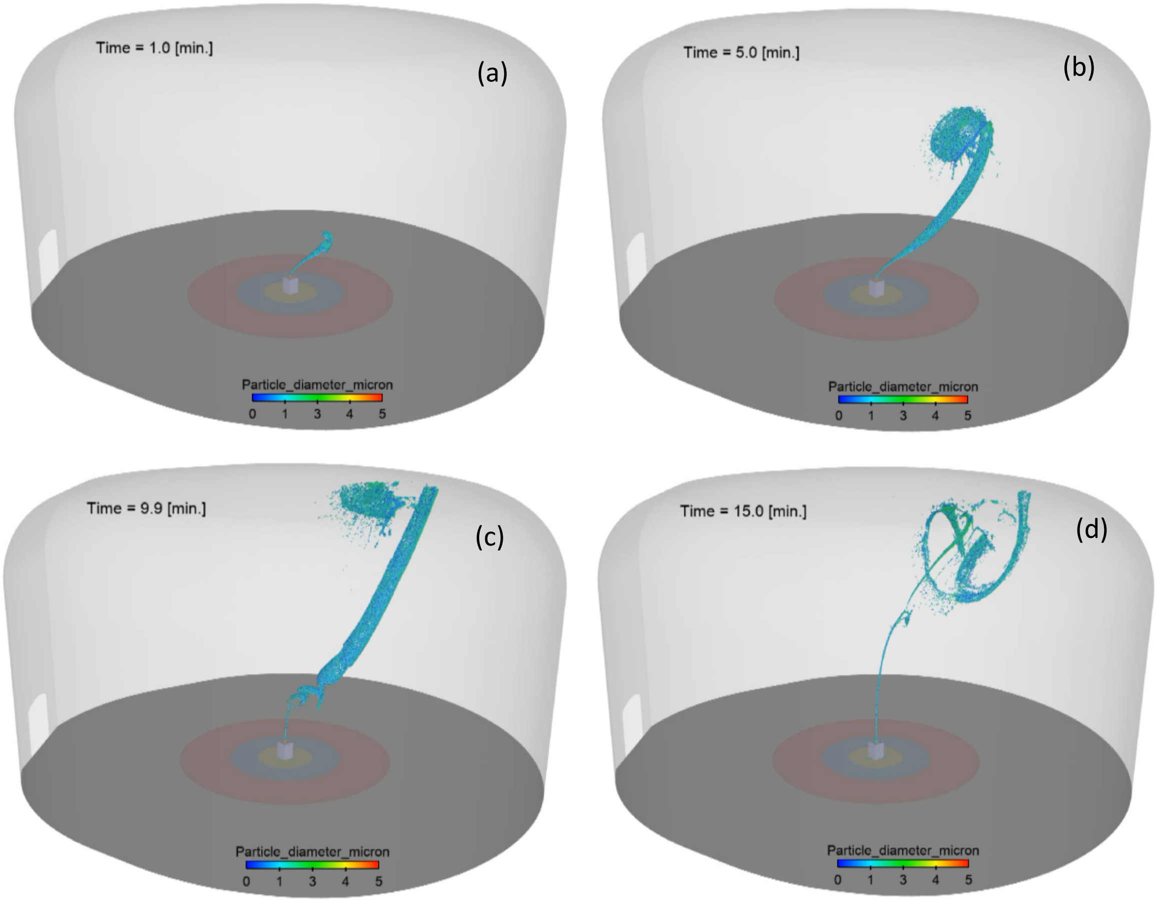

Figure 9. Particle emission from the radius device at (a): 1 minute; (b): 5 minutes; (c) ~10 minutes; (d): 15 minutes. Particles reach the top boundary and escape the domain

However, despite the broader spread, an interesting observation emerges regarding the escape dynamics of the particles. It is noteworthy that the majority of these particles predominantly escape from the top of the simulation dome, rather than from the sides. This suggests a pronounced upward motion of the particles, potentially influenced by the generation of upward air currents within the simulation domain. Consequently, the anticipated spatial extent of particle dispersion beyond the immediate vicinity of the device is expected to be limited under static conditions. Increasing air speed only minimally would greatly increase horizontal flow.

It is also worth noting that no particle deposition is detected on the ground, but rather on the table surface where the device is placed as depicted in figure 10. This is due to the fact that particles released from the device are small, and they have a tendency to remain airborne and disperse rather than settling on the ground. However, it is important to note that the deposited mass on the table surface is negligible, accounting for only 0.02 % of the total formula mass

released over a 15-minute period. This suggests that the majority of the particles is not depositing on surfaces, but rather remaining in the air.

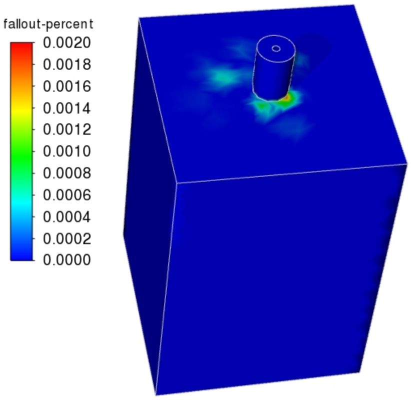

Figure 10. Local deposited mass (fallout) percent on the table surface after 15 minutes of particle emission from the radius device. The total deposited mass is 0.02 % of the total formula emitted into air

- *Settling time of the particle emissions*

In addition to understanding how particles behave in air and the amount of mass they deposit, it is also possible to estimate their airborne duration by calculating their settling velocities. Settling velocity refers to the speed at which a particle falls through air under the influence of gravity. It is the terminal velocity reached by the particle when the drag force acting on it equals the gravitational force pulling it downward, formulated as follows:[8]

$$\mathrm{U} = \frac{d_p^2 g(\rho_p - \rho_a)}{18\eta} \qquad (4)$$

where

U: settling velocity

d_p: particle diameter

g: gravitational acceleration

ρ_p: density of particle

ρ_a: density of air

η: viscosity of air

Knowing the relative distance between the particle and the ground, settling time can be estimated as:

$$t = \frac{H}{U} \qquad (5)$$

where

t: settling time

H: relative distance between particle and the ground

U: settling velocity

It was observed from the simulation that the particles can reach the top boundary of the simulation domain. Therefore, we assumed that H represents the vertical distance between the ground and the top boundary, which is measured to be 15 m.

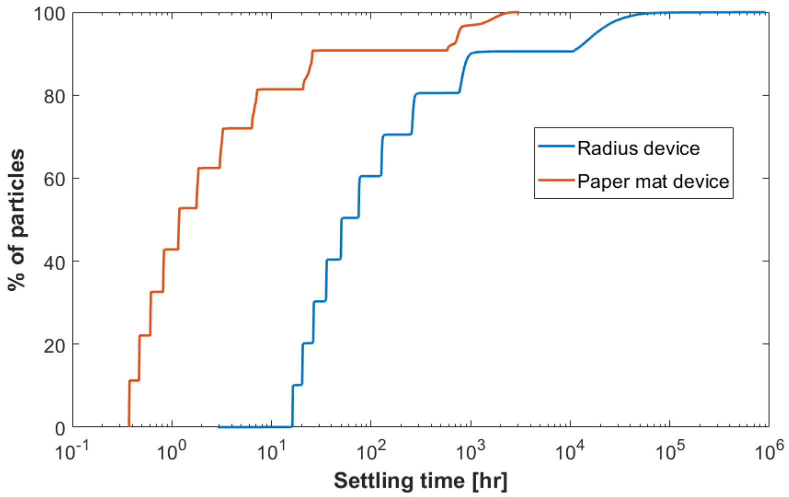

Figure 11. Predicted settling time of the particles from radius and paper mat devices

It is evident from figure 11 that particles emitted from the radius device have the ability to remain suspended in the air for extended periods. The majority of these particles exhibit airborne longevity of 12 hours or more, which can be attributed to their small size. Additionally, the process of evaporation further diminishes the size of these particles while they are airborne, resulting in even longer settling times. Consequently, it is possible for particles to evaporate entirely before ever reaching the ground, leading to their disappearance in the atmosphere. While it is true that a small fraction of the particles does eventually settle, as mentioned earlier, it is important to note that these particles are primarily found on the table where the device is located.

Figure 11 also indicates that the particles generated by the paper mat device have shorter settling times compared to the particles produced by the radius device. According to the calculation, it is predicted that around 50% of these particles will settle within approximately one hour. However, it is important to note that this calculation assumes constant particle sizes throughout their settling times, which may not hold true for liquid particles. Unlike solid particles, liquid particles undergo changes in size due to surface evaporation, which is controlled by their physicochemical properties and environmental conditions. Consequently, while the calculation offers valuable insights into the particles' touch down times, it tends to

underestimate the actual settling times due to these dynamic factors. Moreover, the simulation results have indicated that the vertical rise of the particles is unlikely to be affected significantly by the sluggish wind. This suggests that if the particles descend before fully evaporating, they are likely to be found in the vicinity of the device.

The extended period of time that the particles remain airborne is significant in terms of the breakdown of the active ingredients. The DT50 for prallethrin in air is 38 minutes whereas the DT50 for metofluthrin in air is 1 hour 44 minutes.[9]

Conclusions

Based on the simulations conducted on particle emissions from the radius and paper mat devices, the following conclusions can be drawn. First, due to their small sizes, the particles have the ability to remain suspended in the air for prolonged periods, especially those emitted from the radius device. This indicates that the particles have high potential for atmospheric persistence and, given breakdown rates in the air, will not persist in their active form. Lastly, deposition of particles can only be observed on the device surface or surface that is close to the device under static conditions, and the amount of particle deposition is relatively insignificant compared to the total mass of the formula released.

References

1. J.C. Stevenson, L. Simubali, T. Mudenda, E. Cardol, U.R. Bernier, A.A. Vazquez, P.E. Thuma, D.E. Norris, M. Perry, D.L. Kline, L.W. Cohnstaedt, P. Gurman, S. D'hers, and N. Elman, Controlled release spatial repellent devices (CRDs) as novel tools against malaria transmission: a semi-field study in Macha, Zambia, Malaria Journal, 2018, 17:437
2. U.R. Bernier, D.L. Kline, A. Vazquez-Abad, M. Perry, L.W. Cohnstaedt, P. Gurman, S. D'hers, and N.M. Elman, A combined experimental-computational approach for spatial protection efficacy assessment of controlled release devices against mosquitoes (Anopheles), PLOS Neglected Tropical Diseases, 2019, 13(3):e0007188
3. E.L. Siegel, M. Olivera, E.M. Roig, M. Perry, A.Y. Li, S. D'hers, N.M. Elman, and S.M. Rich, Spatial repellents transfluthrin and metofluthrin affect the behavior of Dermacentor variabilis, Amblyomma americanum, and Ixodes scapularis in an in vitro vertical climb assay, PLoS ONE, 2022, 17(11):e0269150
4. L. Wang, X. Zheng, S. Stevanovic, Z. Xiang, J. Liu, H. Shi, J. Liu, M. Yu, and C. Zhu, Characterizing pollutant emissions from mosquito repellents incenses and implications in risk assessment of human health, Chemosphere, 2018, 191:962-970
5. Esmen and Corn, Residence Time of Particles in Urban Air, Atmospheric Environment, 1971, vol 5, issue 8: 571-578
6. Ansys Inc. (2021), Ansys Fluent Theory Guide, Ansys Inc.

7. S.G. Bradbury (2021), *Determination of ETOC deposition in a simulated outdoor environment from Thermacell Repellents Inc.'s Thermacell Anti Mosquito II*, EcoSafe Natural Products Inc.
8. P.J. Lloyd (2001), Particle size analysis. In R.A. Meyers (Eds.), Encyclopedia of Physical Science and Technology (pp. 649-654), Academic Press
9. Data from Sumitomo Chemical Company. Confidential.

<u>Appendix:</u>

Determination of ETOC deposition in a simulated outdoor environment from Thermacell Repellents Inc.'s Thermacell Anti Mosquito II

Adobe Acrobat Document
