# Supplementary material for: Airborne metofluthrin, a pyrethroid repellent, does not impact foraging honey bees
Source: J Insect Sci. 2024 Oct 23;24(5):7. doi: 10.1093/jisesa/ieae103 (PMC11497606; doi:10.1093/jisesa/ieae103)
Supplement: ieae103_suppl_Supplementary_Material [file ieae103_suppl_supplementary_material.zip › SI Randomization.docx]

| June 9 | GAMMA |
| --- | --- |
| June 15 | GAMMA |
| June 28 | DELTA |

Randomization results for blue feeder treatment. The yellow feeder was allocated the opposite treatment in each case.

| Treatment | Trial | N |
| --- | --- | --- |
| DELTA | 1 | 30 |
| GAMMA | 1 | 31 |
| DELTA | 2 | 19 |
| GAMMA | 2 | 21 |
| DELTA | 3 | 18 |
| GAMMA | 3 | 13 |
